# Development of a *Plasmodium vivax* malaria model for evaluating the effects of control strategies on the malaria burden in Democratic People’s Republic of Korea

**DOI:** 10.3389/fpubh.2024.1423004

**Published:** 2024-08-22

**Authors:** Hye Seong, Jiyeon Suh, Jun Yong Choi, Jeehyun Lee, Joon-Sup Yeom

**Affiliations:** ^1^Department of Internal Medicine and AIDS Research Institute, Yonsei University College of Medicine, Seoul, Republic of Korea; ^2^School of Mathematics and Computing (Computational Science and Engineering), Yonsei University, Seoul, Republic of Korea; ^3^School of Mathematics and Computing, Yonsei University, Seoul, Republic of Korea

**Keywords:** *vivax* malaria, mathematical modeling, RDT, tafenoquine, DPRK

## Abstract

**Background:**

*Plasmodium vivax* malaria has been one of the most troublesome diseases in the Democratic People’s Republic of Korea (DPRK). Given that a majority of malaria cases are concentrated near the demilitarized zone, concerted elimination efforts from both the Republic of Korea (ROK) and DPRK are essential for a malaria-free Korean Peninsula. This study assessed the impact of rapid diagnostic tests (RDTs) and tafenoquine on malaria incidence in DPRK.

**Methods:**

We patterned the current model structure from the previously developed *Plasmodium vivax* malaria dynamic transmission model for ROK. Model parameters were adjusted using demographic and climate data from malaria-risk areas in DPRK, and the model was calibrated to annual malaria incidences from 2014 to 2018 in DPRK, as reported by the World Health Organization. Subsequently, we estimated the preventable malaria cases over a decade after introducing RDTs and tafenoquine compared to using microscopy alone and primaquine, respectively. Sensitivity analysis was performed to account for uncertainty in model parameters.

**Results:**

When comparing RDTs to microscopy, a one-day reduction in diagnostic time due to the introduction of RDTs led to a reduction in malaria incidence by 26,235 cases (65.6%) over the next decade. With a two-day reduction, incidences decreased by 33,635 (84.1%). When comparing a single dose of tafenoquine with a 14-day primaquine regimen, the former prevented 1,222 (77.5%) relapse cases and 4,530 (11.3%) total cases over the years.

**Conclusion:**

The continuous and simultaneous implementation of RDTs and tafenoquine emerges as a potent strategy to considerably reduce malaria in DPRK.

## 1 Introduction

*Plasmodium vivax* malaria was successfully eradicated from the Korean peninsula in the 1970s but resurged in both the Republic of Korea (ROK) and Democratic People’s Republic of Korea (DPRK) during the 1990s (1). ROK detected its first resurgence among soldiers near the Demilitarized Zone (DMZ) in 1993 and promptly initiated measures to control its spread (2). In contrast, DPRK encountered a more formidable challenge. Even with the international malaria control initiatives providing diagnosis, treatment, and preventive measures, the incidence rate of malaria in DPRK remains notable (3, 4).

The DMZ and its surrounding regions have become major hotspots due to the re-emergence of *Plasmodium vivax* malaria (5). The geographical closeness to the DMZ of the malaria-endemic areas in DPRK, coupled with the parallel malaria patterns observed between the two Koreas, emphasizes that efforts from a single side are insufficient. A combined approach from both nations is essential to achieve a malaria-free Korean Peninsula (4, 6).

Given the persistence of malaria in DPRK, there is an increased emphasis on modern diagnostic and treatment tools. The introduction of rapid diagnostic tests (RDTs) is expected to revolutionize early detection, promising timely intervention, and a prospective reduction in transmission (7). Concurrently, the use of tafenoquine, a novel antimalarial agent, offers potential not just in treatment but also in halting the disease’s spread (8).

In this study, we use a dynamic mathematical model to assess the potential impact of modern malaria control strategies, particularly RDTs and tafenoquine, on malaria prevalence in the DPRK. This study is envisioned to provide evidence to guide the Korean peninsula toward a malaria-free future.

## 2 Methods

### 2.1 Study design and data sources

This study adopted the model structure from a previous research (9), with the model parameter values adjusted using data specific to DPRK. For human demographic parameters, the birth and death rates of the DPRK population were obtained from the World Bank statistics (10, 11), and for seasonal mosquito parameters, daily temperature of DPRK was obtained from the Korea Meteorological Administration (12). Yearly incidence data between 2014 and 2018 reported by WHO were used for model fitting (13). After model calibration, we designed and simulated diagnosis and treatment intervention scenarios, each with different infectious period and relapse probability based on the previous studies (9, 14). We estimated the effectiveness of RDT and tafenoquine by predicting the malaria incidence over the next 10 years and comparing relapse and total incidence.

### 2.2 Modeling

**Figure 1:**
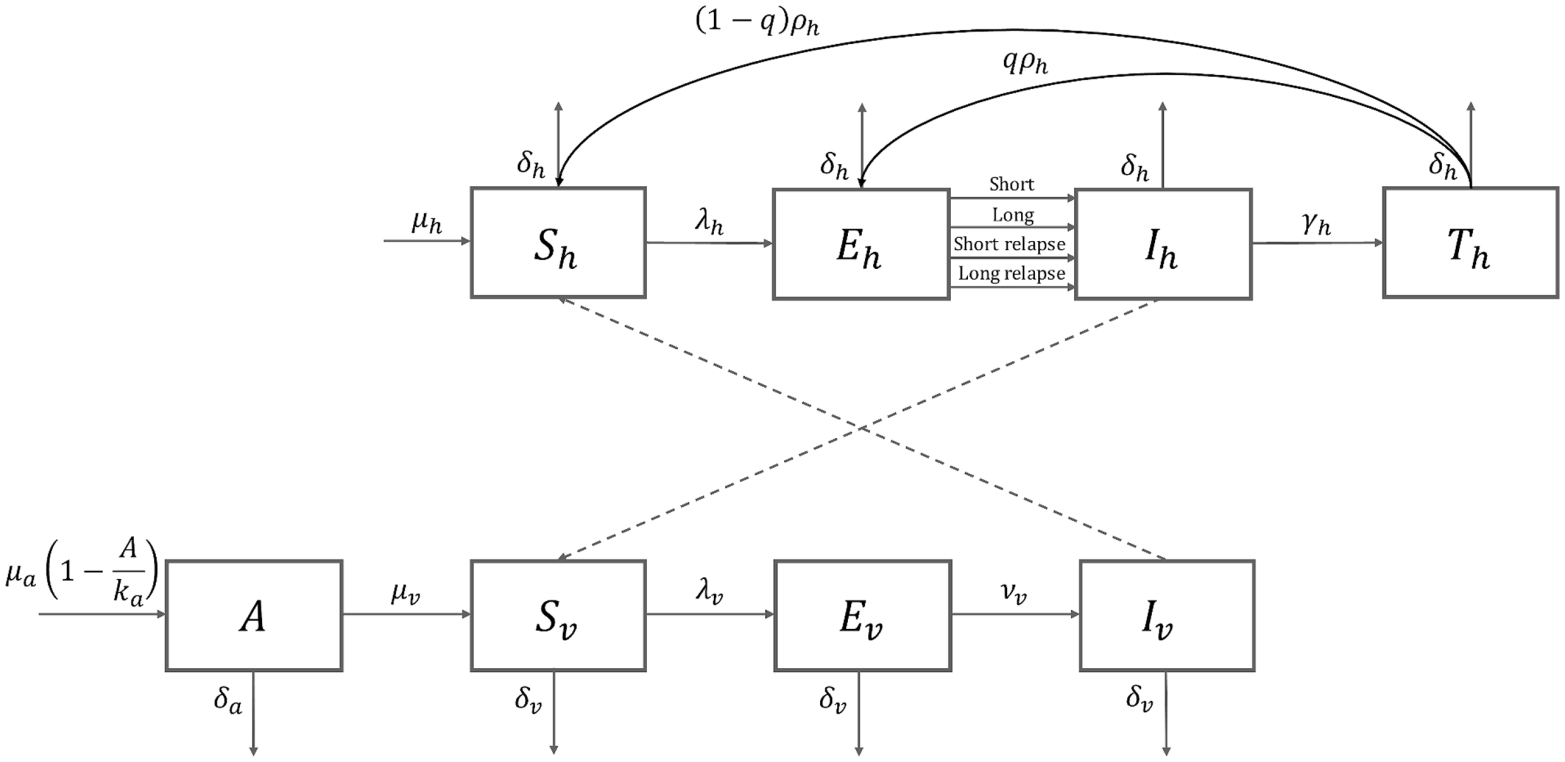
*Plasmodium vivax* malaria transmission model diagram. *S*_*h*_, susceptible human, *E*_*h*_, exposed human, *I*_*h*_, infectious human, *T*_*h*_, treated human, *A*, aquatic stage (immature mosquito), *S*_*v*_, susceptible mosquito, *E*_*v*_, exposed mosquito, *I*_*v*_, infectious mosquito.

The model involved compartments for humans and mosquitoes, and each population was further divided according to malaria infection and progression (Figure 1). Specific model equations are presented in the supplementary information. Model parameter values were based on DPRK’s demographic, climate, and epidemiological data, and listed in Supplementary Table S1 with descriptions and references. Three parameters related to the malaria transmission, p , $\beta_{hv}$ , and $\beta_{vh}$ , were estimated from the yearly incidence data from 2014 to 2018 in DPRK using maximum likelihood estimation. The estimates are also included in Supplementary Table S1, and the incidence data and model prediction from the estimates are shown in Supplementary Figure S1.

### 2.3 Interventions

To assess the impact of the introduction of RDT, we established a baseline scenario with microscopy diagnosis, assuming a mean infectious period of 5 days ( $1/\gamma_{h}$ ). Since no data were available on the time it takes to diagnose malaria through microscopic examination in DPRK, we made an assumed of 5 days, which is 1 day longer than the average in ROK. Subsequently, we devised four scenarios for RDT implementation, each characterized by a reduction in the mean infectious period of 0.5, 1, 1.5, and 2 days.

To evaluate the impact of tafenoquine introduction, we set primaquine treatment as the base scenario and assumed a relapse probability of 0.04, consistent with our previous studies (9, 14). We then considered three tafenoquine treatment scenarios by decreasing the relapse probability to 0.03, 0.02, and 0.01.

Additionally, we estimated the simultaneous impact of RDT and tafenoquine by combining diagnosis and treatment scenarios. A total of 20 combinations of diagnosis and treatment scenarios were generated and simulated.

### 2.4 Sensitivity analysis

We conducted sensitivity analyses to examine the uncertainties of the model parameters on the results. For each of the RDT, TQ, and RDT + TQ scenarios, changes in the prevented cases (%) according to changes in the following eight parameters were investigated: short latency period, long latency period, short latency period for relapse, long latency period for relapse, infectious period, relapse probability, duration of drug action, and vector carrying capacity. In the one-way sensitivity analysis, we performed probabilistic sensitivity analysis assuming uniform distribution under the given range of each parameter. From the distribution of each parameter, 1,000 samples were drawn, and the prevented cases was computed to generate box plot.

In multi-way sensitivity analysis, we simultaneously sampled all the eight parameter values from each distribution 1,000 times and computed the prevented cases and presented them in a histogram. Furthermore, we projected the 1,000 results onto one parameter axis and fitted a linear line to examine the trend according to this particular parameter under uncertainties of the others.

## 3 Results

### 3.1 Prediction of malaria incidence by scenario

**Figure 2:**
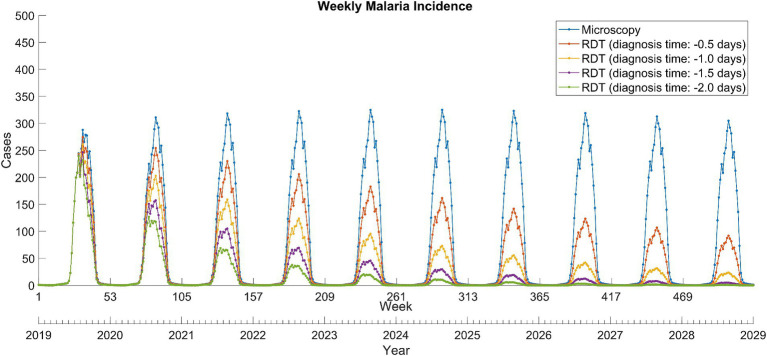
Weekly total *Plasmodium vivax* malaria incidence between 2019 and 2028 in North Korea. RDT, rapid diagnostic test.

Compared to microscopy tests, the introduction of RDTs dramatically reduced the total malaria incidence over the next decade (Figure 2). In the scenario where the diagnosis time was shorten by 2 days, malaria almost disappeared in 2028. The impact of introducing RDTs with reduced diagnosis times varied depending on the scenario, resulting in the percentage prevention of malaria cases over the next 10 years to a range of 43.3–84.1% (Table 1).

**Table 1:** Accumulated total and relapse *Plasmodium vivax* malaria cases over the next decade from 2019 to 2028.

Scenario	Total			Relapse		
	Cases	Prevented cases	Prevented cases (%)	Cases	Prevented cases	Prevented cases (%)
Diagnosis						
RDT (diagnosis time: −2 days)	6,374	33,635	84.1%	324	1,252	79.4%
RDT (diagnosis time: −1.5 days)	9,056	30,953	77.4%	429	1,148	72.8%
RDT (diagnosis time: −1 day)	13,774	26,235	65.6%	610	966	61.3%
RDT (diagnosis time: −0.5 days)	22,701	17,309	43.3%	946	631	40.0%
Microscopy	40,009	–	–	1,577	–	–
Treatment						
TQ (relapse probability: 0.01)	35,479	4,530	11.3%	355	1,222	77.5%
TQ (relapse probability: 0.02)	36,959	3,050	7.6%	734	842	53.4%
TQ (relapse probability: 0.03)	38,538	1,471	3.7%	1,143	433	27.5%
PQ (relapse probability: 0.04)	40,009			1,577		

Prevented cases and percentage were calculated from the difference in cases compared to each base scenario. RDT, rapid diagnostic test; PQ, primaquine; TQ, tafenoquine.

**Figure 3:**
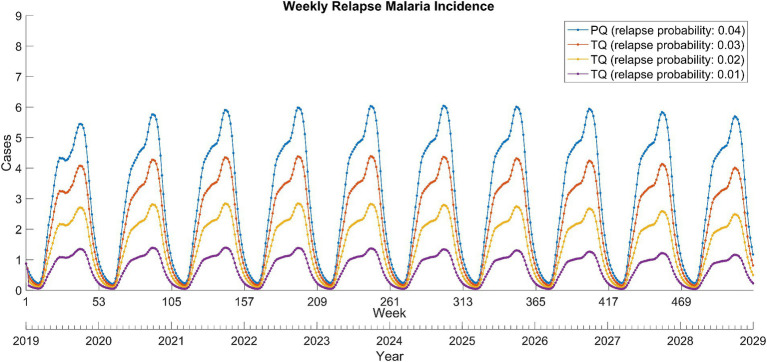
Weekly relapse *Plasmodium vivax* malaria incidence between 2019 and 2028 in North Korea. PQ, primaquine; TQ, tafenoquine.

The replacement of primaquine with tafenoquine resulted in significant reductions in relapse cases (Figure 3). This was proportional to the decrease in relapse probability due to the use of tafenoquine and remained stable over the 10-year period. In addition, the tafenoquine use decreased the total malaria cases by averting secondary infection from relapsed patients (Table 1), and it reduced the total malaria cases by 3.7–11.3% cumulatively over 10 years compared to the primaquine scenario.

**Figure 4:**
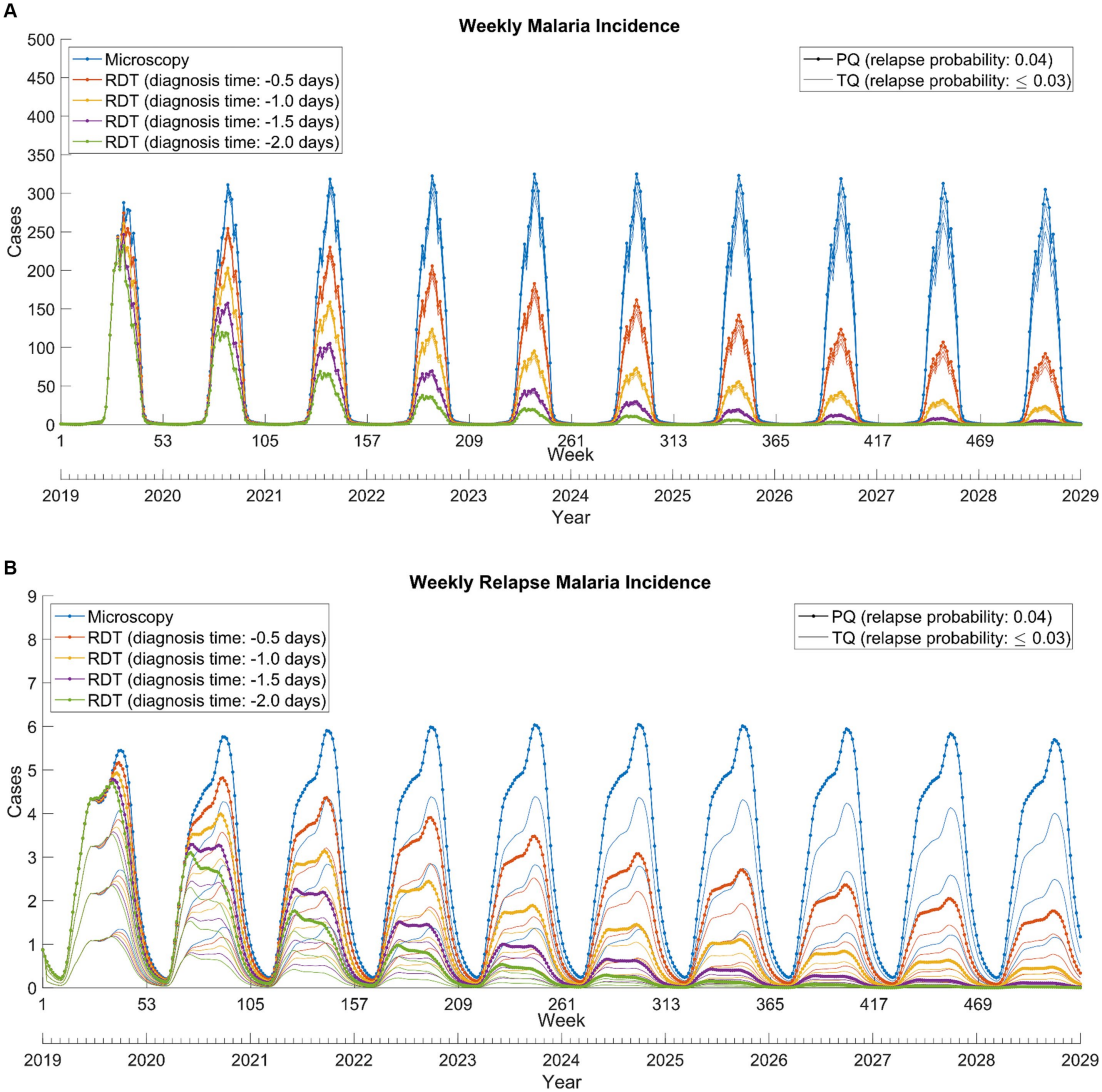
Weekly total **(A)** and relapse **(B)** *Plasmodium vivax* malaria incidence from 2019 to 2028. RDT: rapid diagnostic test, PQ: primaquine, TQ: tafenoquine.

The combination of RDT and tafenoquine scenarios exhibited that RDT was effective in reducing overall cases, whereas tafenoquine was effective in reducing relapse cases upon implementation and further accelerated the reduction in overall cases (Figure 4B). Continuous and concurrent use of RDT and tafenoquine prevented total and relapse malaria cases by up to 85.1 and 95.0%, respectively (Supplementary Table S2).

### 3.2 Sensitivity analysis

#### 3.2.1 One-way sensitivity

Among the four RDT scenarios, we analyzed the sensitivity of the prevented cases (65.6%) in the scenario where the diagnostic time was reduced by 1 day. The long latency period was the most sensitive parameter, followed by the infectious period, vector carrying capacity, and short latency period (Supplementary Figure S2A). The remaining four parameters had minimal effect on the change in the prevented case. Subsequently, we assessed the sensitivity of the prevented case (11.3%) in the tafenoquine scenario where the relapse probability was assumed to be 0.01. In this case, sensitivity to parameters related to relapse, such as relapse probability and long latency period for relapse, increased significantly, while sensitivity to the infectious period, vector carrying capacity, and long latency period remained evident (Supplementary Figure S2B).

Furthermore, a sensitivity analysis was conducted in the RDT and tafenoquine combination scenario. The results were similar to those of the RDT scenario, with the only notable difference being a slight rightward shift in the boxplots due to the tafenoquine implementation (Supplementary Figure S2C).

#### 3.2.2 Multi-way sensitivity

For the multi-way sensitivity analysis, we selected the same RDT scenario with a one-day reduction in diagnosis time as in the one-way sensitivity analysis. Under the simultaneous perturbation of parameters, the distribution of the prevented cases exhibited a slight left skew, with a 95% of uncertainty interval ranging from 18.9 to 80.6% (Supplementary Figure S3A). Projection to the most sensitive parameter, the long latency period, were represented by a scatter plot with curved tendency (Supplementary Figure S3B). Meanwhile, projections to the remaining three sensitive parameters showed scatter plots with linear trend (Supplementary Figures S3C–E). In the tafenoquine scenario with a 0.01 relapse probability, the multivariate sensitivity of the prevented cases displayed a slight right skew, with a 95% uncertainty interval ranging from 6.0 to 20.5% (Supplementary Figure S4A). Meanwhile, projection to the four most sensitive parameters exhibited points spread in a tight and linear line (Supplementary Figures S3B–E). Under the RDT and tafenoquine combination scenario, the results were almost similar to those of the RDT scenario, except for a slight rightward shift attributable to the introduction of tafenoquine. The distribution of the prevented cases was left skewed, and the 95% uncertainty interval ranged from 25.1 to 83.7%.

## 4 Discussion

Our study rigorously assesses the potential effects of RDTs and tafenoquine on *Plasmodium vivax* malaria in DPRK using a mathematical model. The results highlighted the substantial potential of RDTs to reduce malaria incidence, particularly with expedited diagnosis times. Additionally, tafenoquine presents itself as a formidable alternative to primaquine in effectively mitigating relapse cases. Our study validates that a combined application of RDTs and tafenoquine is a formidable strategy, cutting down both total and relapse malaria incidence.

*Plasmodium vivax* malaria, characterized by its unique biological properties, brings forth multiple challenges. The early onset of gametocytemia, which manifests before clinical symptoms, can lead to malaria transmission even before treatment initiation (15). This emphasizes the importance of prompt diagnosis and intervention. Our study underlines the pivotal role of RDTs in addressing the malaria challenge in DPRK, with even a single day’s reduction in diagnosis time significantly reducing transmission (Figure 4A).

The hepatic reservoir in *Plasmodium vivax* leads to relapses, adding complexity to the malaria situation (16). A study from Brazil, where *Plasmodium vivax* represents 91.5% of malaria cases, demonstrated using a transmission dynamics model that tafenoquine could effectively clear hypnozoites and reduce transmission, notably in regions with suboptimal primaquine adherence (17). Our study on DPRK aligns with these insights, underscoring the pivotal role of tafenoquine in the country’s malaria eradication efforts.

Despite DPRK’s inclusion in the WHO’s E-2025 initiative, the nation grapples with a persistent malaria epidemic (1). The halt in support from the Global Fund since 2019 due to the COVID-19 pandemic, precipitated a drop in RDT distribution (1, 13). This reduction in RDT availability aligns with a concerning observation: following a steady decline in malaria cases through 2020, an abrupt increase was observed, with reported cases reaching 2,357 in 2021. This development underscores the vital role of RDTs in prompt malaria diagnosis and management in DPRK, mirroring the central premise of our study.

In our previous study, we developed a *Plasmodium vivax* malaria transmission model for ROK, which evaluated the impacts of introducing RDT and tafenoquine (9, 14). Those simulations revealed that the introduction of these tools significantly curtailed both total and relapse malaria incidence. Drawing parallels from the ROK model, our current study for DPRK similarly underscores the potential benefits of introducing RDTs and tafenoquine. The findings reiterate that their adoption could be pivotal in the efforts to eradicate malaria in DPRK.

Our study offers pivotal insights into malaria control in DPRK using RDTs and tafenoquine; however, it has limitations. The model is based on data available up to 2018, and acquiring comprehensive, recent data from DPRK was challenging, leading to several estimations. The model’s assumptions on consistent uptake of treatments may not mirror real-world scenarios, especially in the wake of the unforeseen challenges of the 2019 COVID-19 pandemic. Furthermore, while tailored for DPRK, the findings might not be generalizable to other regions with differing conditions.

Additionally, due to the difficulty in accessing data on the actual limit of detection of RDT and the prevalence of asymptomatic cases in DPRK, our study only considered symptomatic cases with detectable parasitaemia under microscopic examination or RDT. Including asymptomatic cases could provide a more comprehensive picture of malaria transmission dynamics, and the exclusion of these cases represents another limitation in our model.

In conclusion, our research underscores the imperative need for introducing RDTs and tafenoquine in DPRK. Collaborative efforts between the two nations, guided by these findings, could be the beacon of hope for a malaria-free Korean Peninsula.

## Data Availability

The original contributions presented in the study are included in the article/Supplementary material, further inquiries can be directed to the corresponding authors.

## References

[1] World Health Organization. World malaria report 2021. Genève, Switzerland: World Health Organization (2021).

[2] Korea Centers for Disease Control and Prevention (KCDC). Infectious Diseases Surveillance Yearbook, 2020, Osong, Republic of Korea: KCDC (2021).

[3] Kim H-O Ko T-C Kim S-S Im S-G Kim Y-N. Control of plasmodium vivax malaria by mass chemoprevention with primaquine. Parasitol Open. (2018) 4:e18. doi: 10.1017/pao.2018.13, PMID: 38922648

[4] Sung J Cheong HK Lim AY Kim JH. A small window into the status of malaria in North Korea: estimation of imported malaria incidence among visitors from South Korea. Epidemiol Health. (2020) 42:e2020068. doi: 10.4178/epih.e2020068, PMID: 33227181 PMC8137370

[5] Kim JH Lim AY Cheong HK. Malaria incidence of the regions adjacent to the demilitarized zone in the Democratic People's Republic of Korea, 2004-2016. J Korean Med Sci. (2019) 34:e227. doi: 10.3346/jkms.2019.34.e227, PMID: 31538416 PMC6753370

[6] Lee SK Hu F Firdaus ER Park JH Han JH Lee SE. Surveillance on the vivax malaria in endemic areas in the Republic of Korea based on molecular and serological analyses. Korean J Parasitol. (2020) 58:609–17. doi: 10.3347/kjp.2020.58.6.609, PMID: 33412764 PMC7806437

[7] Maltha J Gillet P Jacobs J. Malaria rapid diagnostic tests in endemic settings. Clin Microbiol Infect. (2013) 19:399–407. doi: 10.1111/1469-0691.12151, PMID: 23438048

[8] Lu KY Derbyshire ER. Tafenoquine: a step toward malaria elimination. Biochemistry. (2020) 59:911–20. doi: 10.1021/acs.biochem.9b01105, PMID: 32073254 PMC8034837

[9] Suh J Kim JH Kim JD Kim C Choi JY Lee J. Cost-benefit analysis of Tafenoquine for radical cure of plasmodium vivax malaria in Korea. J Korean Med Sci. (2022) 37:e212. doi: 10.3346/jkms.2022.37.e212, PMID: 35818703 PMC9274106

[10] The World Bank. Birth rate, crude (per 1,000 people) - Korea, Dem. People's Rep. (2021). Available at: https://data.worldbank.org/indicator/SP.DYN.CBRT.IN?locations=KP (accessed July 20, 2021).

[11] The World Bank. Death rate, crude (per 1,000 people) - Korea, Dem People's Rep (2021). Available at: https://data.worldbank.org/indicator/SP.DYN.CDRT.IN?locations=KP (accessed July 20, 2021).

[12] Korea Meteorological Administration. North Korea meteorological observation data. (2021). Available at: https://data.kma.go.kr/data/grnd/selectNkRltmList.do?pgmNo=58 (accessed July 20, 2021).

[13] World Health Organization. World malaria report 2022. Genève, Switzerland: World Health Organization (2022).

[14] Kim JH Suh J Lee WJ Choi H Kim JD Kim C. Modelling the impact of rapid diagnostic tests on plasmodium vivax malaria in South Korea: a cost-benefit analysis. BMJ Glob Health. (2021) 6. doi: 10.1136/bmjgh-2020-004292 PMC7888375 33593755

[15] Auburn S Cheng Q Marfurt J Price RN. The changing epidemiology of plasmodium vivax: insights from conventional and novel surveillance tools. PLoS Med. (2021) 18:e1003560. doi: 10.1371/journal.pmed.1003560 33891580 PMC8064506

[16] White NJ. Why do some primate malarias relapse? Trends Parasitol. (2016) 32:918–20. doi: 10.1016/j.pt.2016.08.014, PMID: 27743866 PMC5134685

[17] Nekkab N Lana R Lacerda M Obadia T Siqueira A Monteiro W. Estimated impact of tafenoquine for plasmodium vivax control and elimination in Brazil: a modelling study. PLoS Med. (2021) 18:e1003535. doi: 10.1371/journal.pmed.1003535, PMID: 33891582 PMC8064585

