# Supplementary material for: Development of a Plasmodium vivax malaria model for evaluating the effects of control strategies on the malaria burden in Democratic People’s Republic of Korea
Source: Front Public Health. 2024 Aug 22;12:1423004. doi: 10.3389/fpubh.2024.1423004 (PMC11374722; doi:10.3389/fpubh.2024.1423004)
Supplement: Supplementary file 1 [file Data_Sheet_1.docx]

Supplementary Material

**Contents**

1. ***Plasmodium vivax* malaria model equations**
2. **Supplementary Table 1. Model parameters and their descriptions, values, and references**
3. **Supplementary Figure 1. Data and model prediction of annual *Plasmodium vivax* malaria incidence in North Korea between 2014 and 2018.**
4. **Supplementary Table 2. Total and relapse *Plasmodium vivax* malaria cases by diagnosis and treatment scenario**
5. **Supplementary Figure 2. Tornado boxplots for one-way sensitivity analysis by scenario**
6. **Supplementary Figure 3. Histogram and scatter plots for multivariate sensitivity analysis of RDT scenario**
7. **Supplementary Figure 4. Histogram and scatter plots for multivariate sensitivity analysis of tafenoquine scenario**
8. **Supplementary Figure 5. Histogram and scatter plots for multivariate sensitivity analysis of RDT and tafenoquine combination scenario**
9. **References**

## *Plasmodium vivax* malaria model equations

The model was based on delay differential equations (DDEs) and given as follows.

$$\frac{dS_{h}(t)}{dt}=\mu_{h}N_{h}\left( t \right)-\lambda_{h}\left( t \right)S_{h}\left( t \right)+\left( 1-q \right)\rho_{h}T_{h}\left( t \right)- \delta_{h}S_{h}\left( t \right),$$

$$\frac{dE_{h}(t)}{dt}=\lambda_{h}\left( t \right)S_{h}\left( t \right)-p\lambda_{h}\left( t-\tau_{s} \right)S_{h}\left( t-\tau_{s} \right)e^{-\delta_{h}\tau_{s}}-\left( 1-p \right)\lambda_{h}\left( t-\tau_{l} \right)S_{h}\left( t-\tau_{l} \right)e^{-\delta_{h}\tau_{l}}+q\rho_{h}T_{h}\left( t \right)-qp\rho_{h}T_{h}\left( t-\tau_{rs} \right)e^{-\delta_{h}\tau_{rs}}-q\left( 1-p \right)\rho_{h}T_{h}\left( t-\tau_{rl} \right)e^{-\delta_{h}\tau_{rl}}-\delta_{h}E_{h}\left( t \right),$$

$$\frac{dI_{h}\left( t \right)}{dt}=p\lambda_{h}\left( t-\tau_{s} \right)S_{h}\left( t-\tau_{s} \right)e^{-\delta_{h}\tau_{s}}+\left( 1-p \right)\lambda_{h}\left( t-\tau_{l} \right)S_{h}\left( t-\tau_{l} \right)e^{-\delta_{h}\tau_{l}}+qp\rho_{h}T_{h}\left( t-\tau_{rs} \right)e^{-\delta_{h}\tau_{rs}}+q\left( 1-p \right)\rho_{h}T_{h}\left( t-\tau_{rl} \right)e^{-\delta_{h}\tau_{rl}}-\gamma_{h}I_{h}\left( t \right)-\delta_{h}I_{h}\left( t \right),$$

$$\frac{dT_{h}(t)}{dt}=\gamma_{h}I_{h}\left( t \right)-\rho_{h}T_{h}\left( t \right)-\delta_{h}T_{h}\left( t \right),$$

$$\frac{dA(t)}{dt}=\mu_{a}\left( t \right)\left( 1- \frac{A\left( t \right)}{k_{a}} \right)N_{v}\left( t \right)-\mu_{v}\left( t \right)A\left( t \right)-\delta_{a}\left( t \right)A\left( t \right),$$

$$\frac{dS_{v}(t)}{dt}=\mu_{v}\left( t \right)A\left( t \right)-\lambda_{v}\left( t \right)S_{v}\left( t \right)-\delta_{v}\left( t \right)S_{v}\left( t \right),$$

$$\frac{dE_{v}(t)}{dt}=\lambda_{v}\left( t \right)S_{v}\left( t \right)-\nu_{v}E_{v}\left( t \right)-\delta_{v}\left( t \right)E_{v}(t)$$

$$\frac{dI_{v}(t)}{dt}=\nu_{v}E_{v}\left( t \right)-\delta_{v}\left( t \right)I_{v}(t)$$

where $\lambda_{h}\left( t \right)=b\left( t \right)\beta_{hv}\frac{I_{v}}{N_{h}}$, $\lambda_{v}\left( t \right)=b\left( t \right)\beta_{vh}\frac{I_{h}}{N_{h}}$, $N_{h}\left( t \right)=S_{h}\left( t \right)+E_{h}\left( t \right)+I_{h}\left( t \right)+T_{h}(t)$, and $N_{v}\left( t \right)=S_{v}\left( t \right)+E_{v}\left( t \right)+I_{v}(t)$.

## Supplementary Table 1. Model parameters and their descriptions, values, and references

| **Parameter** | **Description** | **Value** | **Reference** |
| --- | --- | --- | --- |
| Humans | | | |
| $\mu_{h}$ | Birth rate of humans | 0.0139 per year  0.0139/365 per day | [1] |
| $\delta_{h}$ | Death rate of humans | 0.0088 per year  0.0088/365 per day | [2] |
| $p$ | Probability of having a short latency period | 0.5330 | Estimated |
| $\tau_{s}$ | Average short latency period | 14 days | [3] |
| $\tau_{l}$ | Average long latency period | 330 days | [3] |
| $\tau_{rs}$ | Average short latency period for relapse | 14 days | [4] |
| $\tau_{rl}$ | Average long latency period for relapse | 274 days | [4] |
| $\gamma_{h}$ | Treatment starting rate = 1/Average infectious period | 1/5 per day | Assumed |
| $\rho_{h}$ | Recovery rate = 1/Average duration of chloroquine action | 1/35 per day | [5] |
| $q$ | Probability of relapse | 0.04 | [3, 6] |
| Mosquitoes | | | |
| $k_{a}$ | Vector carrying capacity | 239 $\times$ $N_{h}$ | Assumed |
| $\mu_{a}$* | Egg deposition rate per adult mosquito  $max\left\{ -0.153T^{2}+8.61T-97.7, 0 \right\}$  $T$: temperature(°C) |  | [7—9] |
| $\delta_{a}$* | Death rate of immature mosquitoes  $\min\left\{ 0.002 exp\left( \left( \frac{T-23}{6.05} \right)^{2} \right), 1 \right\}$ |  | [7, 9, 10] |
| $\mu_{v}$* | Maturation rate  $\left\{ \begin{aligned} \frac{e\left( T \right)p_{E}\left( R \right)p_{L}\left( T,R \right)p_{P}(R)}{\tau_{EA}(T)} 16.5\leq T\leq35.6 \\ 0 \mathrm{Otherwise} \end{aligned} \right.$   - $e\left( T \right)=\frac{f(T)}{\delta_{v}(T)}$: Lifetime number of eggs laid by adult mosquitoes. - $f\left( T \right)=-0.153T^{2}+8.61T-97.7$: Total number of eggs laid per day. - $1/{\delta_{v}(T}$): Average adult mosquito lifespan. - $p_{E}\left( R \right)=\frac{3.6(R_{L}-R)}{R_{L}^{2}}$: Daily survival probability of eggs. $R$ and $R_{L}$ denote rainfall (mm) and rainfall threshold (mm), and fixed to 3mm and 76mm, respectively. - $p_{L}\left( T,R \right)=exp\left\{ -0.00554T+0.06737 \right\}\frac{R(R_{L}-R)}{R_{L}^{2}}$: Daily survival probability of larvae. - $p_{P}\left( R \right)=\frac{3R(R_{L}-R)}{R_{L}^{2}}$: Daily survival probability of pupae. - $\tau_{EA}\left( T \right)=1/\left( -0.00094T^{2}+0.049T-0.552 \right)$: Total development time from egg to adult mosquito. |  | [7, 9, 11] |
| $\delta_{v}$* | Death rate of adult mosquitoes  $\left\{ \begin{aligned} 1 T\leq-4 \\ -\frac{29}{570}T+\frac{227}{259} -4<T\leq15 \\ \frac{1}{30} 15<T\leq32 \\ \frac{29}{570}T-\frac{303}{190} 32<T \end{aligned} \right.$ |  | [3, 7—9] |
| $\nu_{v}$ | Progression rate of mosquitoes to the infectious state = 1/Average latency period of mosquitoes | 1/10 per day | [3, 12] |
| Transmission | | | |
| $\lambda_{h}$* | Force of infection from mosquitoes to humans  $\lambda_{h}\left( T \right)=b_{h}\left( T \right)\beta_{hv}\frac{I_{v}}{N_{v}}=b\left( T \right)\beta_{hv}\frac{I_{v}}{N_{h}}$ |  | [13] |
| $\lambda_{v}$* | Force of infection from humans to mosquitoes  $\lambda_{v}\left( T \right)=b_{v}\left( T \right)\beta_{vh}\frac{I_{h}}{N_{h}}=b\left( T \right)\beta_{vh}\frac{I_{h}}{N_{h}}$ |  | [13] |
| $b_{h}$* | Biting rate for humans is defined as the number of mosquito bites per human per unit time  $b_{h}\left( T \right)=b\left( T \right)\times\frac{N_{v}}{N_{h}}$  $=\max\left\{ 0.000203T\left( T-11.7 \right)\sqrt{42.3-T}\times\frac{N_{v}}{N_{h}}, 0 \right\}$ |  | [7—9, 13] |
| $b_{v}$* | Biting rate for mosquitoes refers to the number of human bites for one mosquito per unit time  $b_{h}\left( T \right)=b\left( T \right)$  $=\max\left\{ 0.000203T\left( T-11.7 \right)\sqrt{42.3-T}, 0 \right\}$ |  | [7—9, 13] |
| $\beta_{hv}$ | Probability of transmission of infection from an infectious mosquito to a susceptible human | 0.1168 | Estimated |
| $\beta_{vh}$ | Probability of transmission of infection from an infectious human to a susceptible mosquito | 0.0218 | Estimated |

* refers to temperature-dependent parameters

**3. Supplementary Figure 1.** **
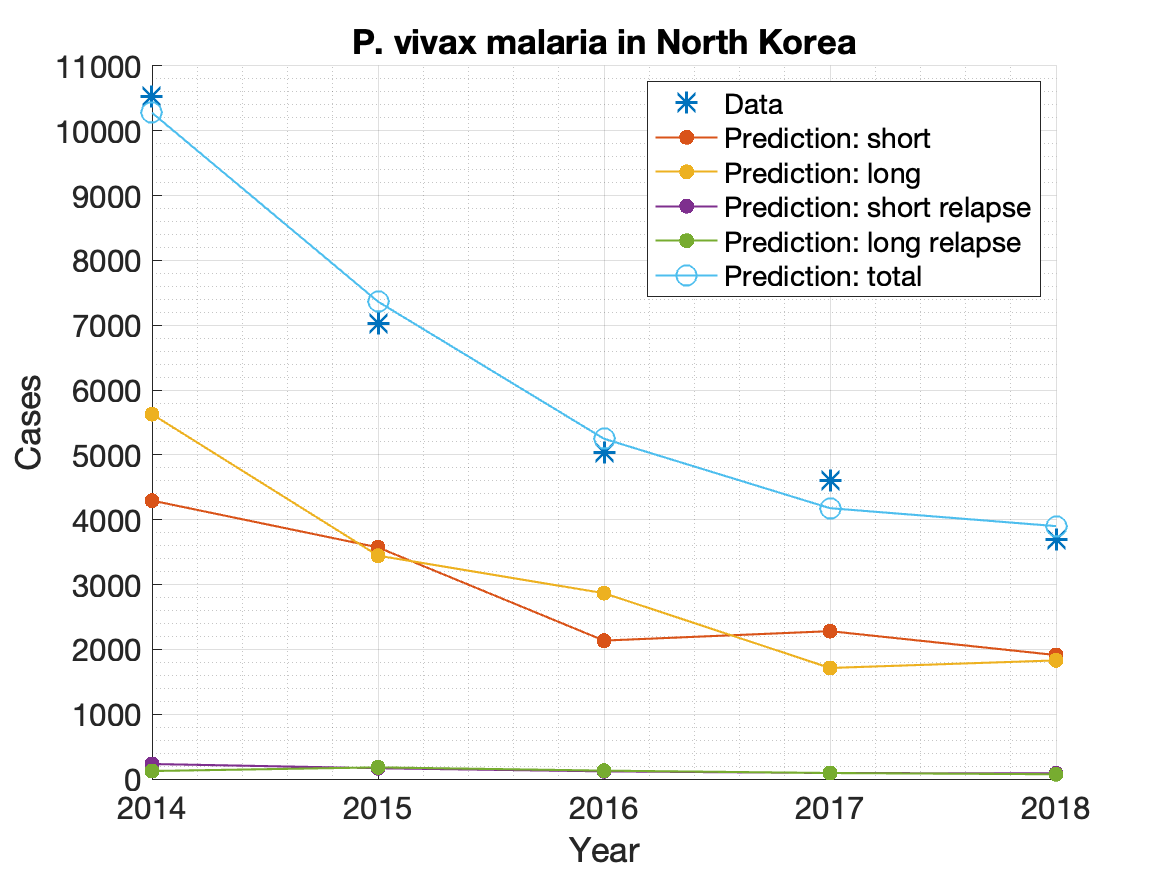
**

**Supplementary Figure 1.** Data and model prediction of annual *Plasmodium vivax* malaria incidence in North Korea between 2014 and 2018.

## Supplementary Table 2. Total and relapse *Plasmodium vivax* malaria cases by diagnosis and treatment scenario

| Scenario | Total | | | Relapse | | |
| --- | --- | --- | --- | --- | --- | --- |
|  | Cases | Prevented  cases | Prevented  cases (%) | Cases | Prevented  cases | Prevented  cases (%) |
| Diagnosis + Treatment | | | | | | |
| RDT (diagnosis time: -2 days) + TQ (relapse probability: 0.01) | 5,967 | 34,042 | 85.1% | 79 | 1,498 | 95.0% |
| RDT (diagnosis time: -2 days) + TQ (relapse probability: 0.02) | 6,110 | 33,899 | 84.7% | 158 | 1,418 | 90.0% |
| RDT (diagnosis time: -2 days) + TQ (relapse probability: 0.03) | 6,229 | 33,780 | 84.4% | 240 | 1,337 | 84.8% |
| RDT (diagnosis time: -2 days) + PQ (relapse probability: 0.04) | 6,374 | 33,635 | 84.1% | 324 | 1,252 | 79.4% |
| RDT (diagnosis time: -1.5 days) + TQ (relapse probability: 0.01) | 8,406 | 31,603 | 79.0% | 103 | 1,474 | 93.5% |
| RDT (diagnosis time: -1.5 days) + TQ (relapse probability: 0.02) | 8,603 | 31,406 | 78.5% | 207 | 1,370 | 86.9% |
| RDT (diagnosis time: -1.5 days) + TQ (relapse probability: 0.03) | 8,824 | 31,185 | 77.9% | 316 | 1,261 | 80.0% |
| RDT (diagnosis time: -1.5 days) + PQ (relapse probability: 0.04) | 9,056 | 30,953 | 77.4% | 429 | 1,148 | 72.8% |
| RDT (diagnosis time: -1 days) + TQ (relapse probability: 0.01) | 12,655 | 27,354 | 68.4% | 143 | 1,433 | 90.9% |
| RDT (diagnosis time: -1 days) + TQ (relapse probability: 0.02) | 13,033 | 26,976 | 67.4% | 292 | 1,284 | 81.5% |
| RDT (diagnosis time: -1 days) + TQ (relapse probability: 0.03) | 13,421 | 26,588 | 66.5% | 448 | 1,128 | 71.6% |
| RDT (diagnosis time: -1 days) + PQ (relapse probability: 0.04) | 13,774 | 26,235 | 65.6% | 610 | 966 | 61.3% |
| RDT (diagnosis time: -1.5 days) + TQ (relapse probability: 0.01) | 20,495 | 19,514 | 48.8% | 217 | 1,359 | 86.2% |
| RDT (diagnosis time: -1.5 days) + TQ (relapse probability: 0.02) | 21,196 | 18,813 | 47.0% | 446 | 1,131 | 71.7% |
| RDT (diagnosis time: -1.5 days) + TQ (relapse probability: 0.03) | 21,922 | 18,087 | 45.2% | 688 | 888 | 56.3% |
| RDT (diagnosis time: -1.5 days) + PQ (relapse probability: 0.04) | 22,701 | 17,309 | 43.3% | 946 | 631 | 40.0% |
| Microscopy + TQ (relapse probability: 0.01) | 35,479 | 4,530 | 11.3% | 355 | 1,222 | 77.5% |
| Microscopy + TQ (relapse probability: 0.02) | 36,959 | 3,050 | 7.6% | 734 | 842 | 53.4% |
| Microscopy + TQ (relapse probability: 0.03) | 38,538 | 1,471 | 3.7% | 1,143 | 433 | 27.5% |
| Microscopy + PQ (relapse probability: 0.04) | 40,009 | - | - | 1,577 | - | - |

RDT: rapid diagnostic test, TQ: tafenoquine, PQ: primaquine.

## Supplementary Figure 2

**A.**

**B.**

**C.**

**Supplementary Figure 2.** Tornado boxplots for one-way sensitivity analysis by scenario. Prevented cases in the A: RDT, B: tafenoquine, and C: RDT and tafenoquine combination scenario. RDT: rapid diagnostic test, PQ: primaquine, CQ: chloroquine.

## Supplementary Figure 3

## A.

**B. C.**

**D. E.**

**Supplementary Figure 3.** Histogram and scatter plots for multivariate sensitivity analysis of RDT scenario. RDT: rapid diagnostic test.

## Supplementary Figure 4

**A.**

**B. C.**

**D. E.**

**Supplementary Figure 4.** Histogram and scatter plots for multivariate sensitivity analysis of tafenoquine scenario. PQ: primaquine.

## Supplementary Figure 5.

**A.**

**B. C.**

**D. E.**

**Supplementary Figure 5.** Histogram and scatter plots for multivariate sensitivity analysis of RDT and tafenoquine combination scenario. RDT: rapid diagnostic test.

## References

[1] The Word Bank, “Birth rate, crude (per 1,000 people) – Korea, Dem. People’s Rep.”, https://data.worldbank.org/indicator/SP.DYN.CBRT.IN?locations=KP, Accessed 2021.07.20.

[2] The Word Bank, “Death rate, crude (per 1,000 people) – Korea, Dem. People’s Rep.”, https://data.worldbank.org/indicator/SP.DYN.CDRT.IN?locations=KP, Accessed 2021.07.20.

[3] Korea Centers for Disease Control and Prevention. “Malaria management guidelines 2019”, https://www.kdca.go.kr/board/board.es?mid=a20507020000&bid=0019&act=view&list_no=143706, Accessed 2021.07.20.

[4] Kwak, Y. G. et al. “Clinical characteristics of *vivax* malaria and analysis of recurred patients”, Infection & Chemotherapy, 45.1 (2013), pp. 69-75

[5] Baird, J. K. “Chloroquine resistance in *Plasmodium vivax*”, Antimicrob. agents and chemotherapy 48, 4075–4083 (2004).

[6] Chu, C. S. & White, N. J. Management of relapsing *Plasmodium vivax* malaria. Expert. review anti-infective therapy 14, 885–900 (2016).

[7] Kim, J. E., Choi, Y. & Lee, C. H. “Effects of climate change on *Plasmodium vivax* malaria transmission dynamics: A mathematical modeling approach”, Applied Mathematics and Computation 347, 616–630 (2019).

[8] Mordecai, E. A. et al. Optimal temperature for malaria transmission is dramatically lower than previously predicted. Ecol. Letters 16, 22–30 (2013).

[9] Korea Meteorological Administration, “North Korea meteorological observation data”, https://data.kma.go.kr/data/grnd/selectNkRltmList.do?pgmNo=58, Accessed 2021.07.20.

[10] Beck-Johnson, L. M. et al. The effect of temperature on anopheles mosquito population dynamics and the potential for malaria transmission. PLOS one 8, e79276 (2013).

[11] Okuneye, K. & Gumel, A. B. Analysis of a temperature-and rainfall-dependent model for malaria transmission dynamics. Math. Biosciences 287, 72–92 (2017).

[12] Mandal, S., Sarkar, R. R. & Sinha, S. “Mathematical models of malaria-a review”, Malaria Journal 10, 1–19 (2011).

[13] Chitnis, N., Cushing, J. M. & Hyman, J. “Bifurcation analysis of a mathematical model for malaria transmission” SIAM Journal on Applied Math. 67, 24–45 (2006).
